# Cucurbitacin I inhibits the proliferation of pancreatic cancer through the JAK2/STAT3 signalling pathway *in vivo* and *in vitro*

**DOI:** 10.7150/jca.65875

**Published:** 2022-03-28

**Authors:** Dongchao Xu, Hongzhang Shen, Mengyao Tian, Wangyang Chen, Xiaofeng Zhang

**Affiliations:** 1 Department of Gastroenterology, Affiliated Hangzhou First People's Hospital, Zhejiang University School of Medicine, Hangzhou310000, China.; 2 Hangzhou Institute of Digestive Diseases, Hangzhou310000, China.; 3 Key Laboratory of Integrated Traditional Chinese and Western Medicine for Biliary and Pancreatic Diseases of Zhejiang Province, Hangzhou310000, China.

**Keywords:** Cucurbitacin I, pancreatic cancer, JAK2/STAT3 signalling pathway, proliferation

## Abstract

Pancreatic cancer is one of the most aggressive solid malignancies, as it has a 5-year survival rate of less than 10%. The growth and invasion of pancreatic cancer cells into normal tissues and organs make resection and treatment difficult. Finding an effective chemotherapy drug for this disease is crucial. In this study, we selected the tetracyclic triterpenoid compound cucurbitacin I, which may be used as a potential therapeutic drug for treating pancreatic cancer. First, we found that cucurbitacin I inhibited pancreatic cancer proliferation in a dose-time dependent manner. Further studies have shown that cucurbitacin I blocks the cell cycle of pancreatic cancer in the G2/M phase and induces cell apoptosis. In addition, under the action of the compound, the invasion ability of cells was greatly reduced and markedly impaired the growth of pancreatic tumour xenografts in nude mice. Furthermore, the decrease in pancreatic cancer cell proliferation caused by cucurbitacin I appeared to involve JAK2/STAT3 signalling pathway inhibition, and the use of JAK2/STAT3 activators effectively restored the inhibition. In conclusion, our research may provide a basis for the further development of pancreatic cancer treatment drugs.

## Introduction

Pancreatic cancer (PDAC) is well known for its high fatality rate, and the incidence of pancreatic cancer has continued to increase in recent years 1. Despite advances in the treatment of pancreatic cancer, survival rates remain poor 1, 2. In the early stage, pancreatic cancer patients usually have no obvious clinical symptoms, and most cancers have metastasized by the time they are discovered 3. Chemotherapy is the main treatment or sometimes the only treatment for pancreatic cancer patients who are in the advanced stage of cancer and can no longer undergo surgical treatment. There are still many problems with clinical medications, and the foremost is the problem of drug resistance 4. Gemcitabine has become the “gold standard” in the treatment of locally advanced and metastatic pancreatic cancer. Pancreatic cancer can easily become resistant to gemcitabine, and most patients begin to develop resistance within a few weeks after treatment, which is an important factor causing the short survival times of patients 5. Therefore, the exploration and discovery of new pancreatic cancer chemotherapy drugs or drug combinations are important endeavours in clinical and scientific research.

Cucurbitacins are a class of tetracyclic triterpenoids isolated from Cucurbitaceae, Cruciferae and other plants 6, 7. Cucurbitacins can be divided into 17 subtypes from A to S according to the derivatives of functional groups and side chains 6. Cucurbitacins have been found to have many biological activities, such as anti-inflammatory activities, anti-oxidationimmunity improvement, and gastrointestinal function activities 8-12. Cucurbitacin I (CuI) is one of cucurbitacin compounds. Previous studies have shown that CuI can protect cardiomyocytes in different ways, including by inhibiting the connective tissue growth factor and protecting against mitochondrial dysfunction 13, 14. In recent years, the anti-cancer effect of CuI has become the focus of researchers. Studies have found that cucurbitacin I inhibits the survival of non‑small cell lung cancer cells by inhibiting the Akt signalling pathway 15. In addition, cucurbitacin I induces protective autophagy in glioblastoma 16.

The results of the present study revealed that cucurbitacin I significantly inhibited the proliferation of four human pancreatic cancer cell lines. The growth inhibitory effect of this compound was related to obvious G2-M phase arrest and an increase in apoptosis by the inhibition of JAK2 and STAT3 and subsequent activation of the caspase cascade. To further confirm our results *in vitro*, we demonstrated that cucurbitacin I provided profound anti-tumour activity in mouse xenograft models with no obvious toxicity to the animals.

## Materials and methods

### Cell culture and reagents

Human pancreatic cancer cell lines (ASPC-1, BXPC-3, CFPAC-1, SW 1990) were purchased from American Type Culture Collection (ATCC). All cell lines used in this study were within 20 passages after receipt. Cell lines were maintained in RPMI-1640 (Sigma-Aldrich). RPMI 1640 medium was supplemented with 10% foetal bovine serum (FBS; Gibco), 100 U/mL penicillin, and 100 µg/mL streptomycin (Sigma-Aldrich). All cells were then incubated at 37 °C in a humidified atmosphere of 5% CO_2_. CuI (Push Biotechnology) was dissolved in dimethyl sulfoxide (DMSO; Sigma-Aldrich) as a 100 mM stock solution and stored at -20°C. Colivelin, a JAK2/STAT3 activator, was purchased from MedChemExpress (MCE).

### Cell proliferation and colony formation assays

Pancreatic cancer cells (3×10^3^ cells/well) were seeded in 96-well plates, incubated overnight in complete RPMI 1640 medium containing 10% FBS, and treated with increasing doses of the respective vehicle control (DMSO) or CuI. Cell viability was measured by Cell Counting Kit-8 (CCK8) after 24, 48 or 72 h. For colony formation assays, we followed the methods of Dandawate et al. 2020 17. A total of 750 viable PDAC cells were seeded in six-well dishes and grown for three days. PDAC cells were then treated with increasing doses of CuI. The culture medium was replaced after 24 h, and CuI exposure was removed. The cells were further incubated for 7 days to ensure that the clones were large enough. The colonies obtained were washed with PBS and fixed using 4% Paraformaldehyde Fix Solution at room temperature, washed with PBS and stained with Crystal Violet Staining Solution.

### EdU staining assay

A commercially available EdU (5-ethynyl-2'-deoxyuridine) staining kit (Beyotime) was used for cell proliferation assays, and all steps were performed in accordance with the manufacturer's instructions. EdU with the fluorescently labelled small molecule azide Alexa Fluor 488 was added to medium containing different concentrations of cucurbitacin I. Nuclei were counterstained with Hoechst. The proportions of EdU-positive cells were used to evaluate the proliferation ability.

### Cell migration assay

The wound healing assay and the Transwell assay were used to measure cell migration. PDAC cells (1×10^6^ well) were seeded into six-well plates and incubated, and the cell confluence was checked to be between 90% and 100%. Serum was used to starve the cells for 24 h and a plastic tip was used to scrape P200. Plates were washed with PBS to remove suspended cells. PDAC cells were incubated with serum-free medium containing different concentrations of CuI. The wound was recorded at 0 h and 24 h under a phase contrast microscope. ImageJ software was used to calculate the cell migration distances.

Transwell insert chambers (Corning) with 8 μm pore filters were used for the Transwell migration assay. Serum-free cell suspensions (cell=5×10^4^) containing different concentrations of CuI were seeded in the upper chambers. The lower chamber was filled with medium containing 20% FBS as a chemoattractant. After incubation for 24 h at 37 °C, the cells in the upper chamber were completely removed by cotton swabs, and the cells in the lower chamber were fixed in 4% PFA for 30 min at room temperature and stained with a crystal violet solution for 30 min at room temperature. After being dried, the number of stained cells was counted.

### Cell invasion assays

Transwell insert chambers with 8 μm pore filters coated with 0.5 mg/mL Matrigel (BD Sciences) were used for the Transwell invasion assay. Briefly, serum-free cell suspensions (cell=5×104) containing different concentrations of CuI were seeded in the upper chambers. The lower chamber was filled with medium containing 20% FBS as a chemoattractant. After incubation for 24 h at 37 °C, the cells in the upper chamber were completely removed by cotton swabs, and the cells in the lower chamber were fixed in 4% PFA for 30 min at room temperature and stained with a crystal violet solution for 30 min at room temperature. After the cells were dried, the number of stained cells was counted.

### Cell cycle analyses

Cell cycle analysis was performed using propidium iodide (PI) staining (BD Sciences) for DNA quantitation. We followed the methods of Chen et al. 2020 18. Pancreatic cancer cells (9×10^4^ cells/well) were seeded in six-well dishes and treated with different concentrations of CuI for 24 h. Cells were harvested, washed and centrifuged at 1000 r/min for 5 min. The cells were suspended in a PBS mixture containing 0.1% Triton X-100 (Sigma-Aldrich), 1 mg/ml propidium iodide (PI, Sigma-Aldrich), and 2 μg DNase-free RNase (Sigma-Aldrich) for 30 min in the dark at room temperature. Flow cytometry was measured with a FACSCalibur analyser (Becton Dickinson), capturing at least 1×10^4^ cells for each measurement. The cell cycle data were processed using Cell Quest Pro software.

### Apoptosis assays

Cell apoptosis was assessed by Annexin V-FITC and propidium iodide staining (BD Sciences). Briefly, 9×10^4^ pancreatic cancer cells/well were plated in six-well dishes overnight and treated with different concentrations of CuI for 24 h. The treated cells were washed with PBS, collected, and stained with Annexin V antibody conjugated with a FITC fluorophore and PI in the dark at room temperature. For each measurement, at least 1×10^4^ cells were analysed on an Accuri C6 flow cytometer (Becton Dickinson) using CFlow Plus software.

### Western blot analysis

For the western blot analysis, pancreatic cancer cells were washed with PBS and lysed in RIPA buffer (Sangon Biotech) containing Protease & Phosphatase Inhibitor (Thermo Fisher). Thirty-microlitre samples were loaded into SDS-polyacrylamide gel electrophoresis (PAGE) gels. Proteins were separated by electrophoresis and transferred to PVDF membranes (Millipore). Membranes were blocked with 3% bovine serum albumin (BSA) at room temperature for 1 h and incubated with a primary antibody overnight at 4 °C. On the second day, the cells were washed three times with TBST and incubated with the secondary antibody at room temperature. The specific proteins were detected by an enhanced chemiluminescence detection system (ECL; Fdbio Science). Protein expression was captured by the Bio-Rad ChemiDoc-XRS + instrument.

### Immunohistochemistry

For the immunohistochemical staining, antigen retrieval and section staining methods were followed as described by Xu et al. 2018 19. Briefly, the tissue samples were cut into sections and washed in xylene to remove the paraffin. Then, the slices were rehydrated by serial dilutions of alcohol and heated in citrate buffer for antigen retrieval. The sample was incubated with the antibody overnight at 4 °C, washed 3 times in PBS the next day, and incubated with the secondary antibody at room temperature for 1 h. The ABC peroxidase method was used to develop colour. The cells were counterstained with haematoxylin and observed under a microscope.

### Xenograft experiments

Five-week-old immune-deficient nude mice (BALB/c-nu) were obtained from Shanghai SLAC Laboratory Animal Company to generate pancreatic cancer mouse models. All the mice were maintained and monitored in a specific pathogen-free environment. The protocol for the experiment was approved, and animals were handled according to the ethical standards of the Institutional Animal Care and Use Committee of Zhejiang Chinese Medical University, complying with the rules of Regulations for the Administration of Affairs Concerning Experimental Animals (approved by the State Council of China, No. SYXK (Zhejiang) 2018-0012). BXPC-3 cells (2×10^6^ cells) were injected into the right abdomen of each nude mouse. After two weeks, 18 nude mice with the same tumour size were selected and randomly divided into 3 groups. Normal saline and a low (1 mg/kg) and a high dose (2 mg/kg) of cucurbitacin I were injected every three days, and the tumour weights and volumes were measured. The formula was as follows: volume = 0.5 × length × width^2^. Thirty days later, the mice were sacrificed, and the tumours were stripped.

### Statistical analyses

All experiments were performed at least three times. Data were analysed by SPSS 12.0 and expressed as the means ± SD. Statistical comparisons between two groups were made using an unpaired Student's t-test, and probability values (p) < 0.05 were considered significant.

## Results

### Cucurbitacin I inhibited the viability of pancreatic cancer cells

**Figure 1:**
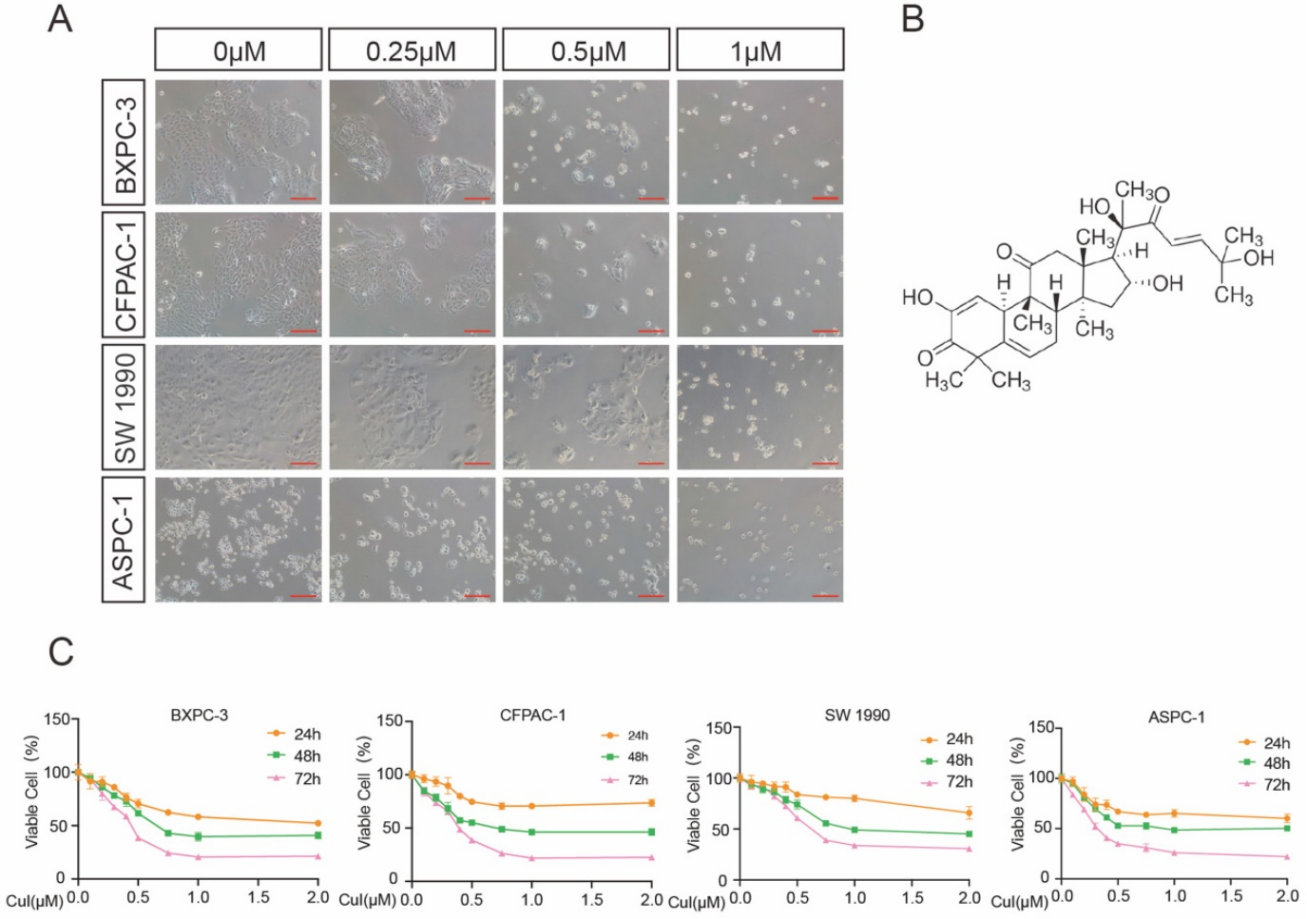
** CuI inhibited PDAC cell viability in a time-dose dependent manner. (A)** Morphological changes of four PDAC cell lines (AsPC-1, BXPC-3, CFPAC-1, SW 1990) treated with different concentrations of CuI (0, 0.25, 0.5 and 1 µM), scale bar: 200 µm. **(B)** Chemical structure of CuI. **(C)** The cell viabilities were measured using the CCK-8. The line graph represents the percentage of viable cells in the control group.

We first examined the effects of CuI on four pancreatic cancer cell lines (AsPC-1, BXPC-3, CFPAC-1, SW 1990). After 72 h of treatment with CuI, cell viability was significantly inhibited, and the cell morphology was also significantly changed. The untreated cells had unique round or polyhedral shapes, and the cells shrank and lost their original shapes after a high-dose compound treatment (Figure 1A). CCK-8 was used to quantify the effect of CuI on cell viability. We observed that the viability of pancreatic cancer cells decreased gradually with increasing CuI doses. In addition, the effects of CuI on the viability of pancreatic cancer cells were detected at 24, 48 and 72 h, and it was found that the longer the compound treatment time was, the weaker the cell activity (Figure 1C). IC 50 values ranged from 0.2726 μM to 0.4842 μM for different cell lines (ASPC-1:0.2726 μM, BXPC-3:0.3852 μM, CFPAC-1:0.3784 μM, SW 1990:0.4842 μM) after 72 h of treatment. Therefore, we concluded that CuI inhibited pancreatic cancer cell viability in a time-dose dependent manner.

### Cucurbitacin I inhibited the proliferation of pancreatic cancer cells

**Figure 2:**
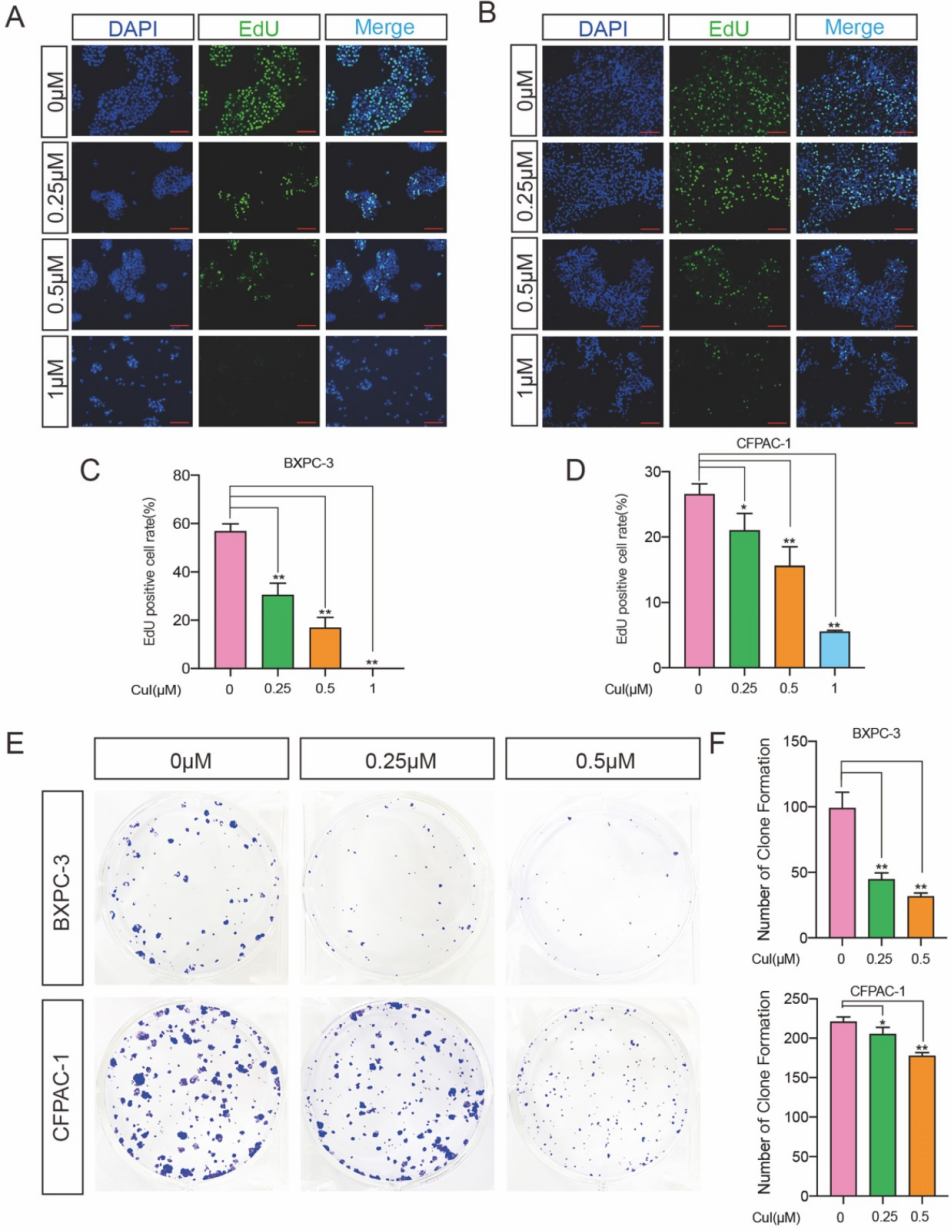
** CuI inhibited the proliferation of PDAC cells. (A-B)** Detection by fluorescence microscopy of EdU (green) incorporated into the DNA of cultured BXPC-3 and CFPAC-1cells, scale bar: 200 µm. The nuclei were counter-stained with DAPI (blue). **(C-D)** The EdU positive rate of BXPC-3 and CFPAC-1 cells. **(E)** The colony formation assays of BXPC-3 and CFPAC-1 cells treated with different concentration (0-0.5 µM) of CuI. **(F)** The number of clones of BXPC-3 and CFPAC-1 cells.

DNA synthesis is a crucial step in the process of cell proliferation. To further determine the effect of CuI on the proliferation of pancreatic cancer cells, we used EdU cell proliferation assays to detect the effect of the compound on DNA synthesis. Two pancreatic cancer cell lines, BXPC-3 and CFPAC-1, were used for detection, and it was found that both the percentage of BXPC-3- and CFPAC-1-positive cells were significantly reduced (Figure 2A-D). Similar results also appeared on ASPC-1 and SW 1990 (Supplementary Figure 1). We next performed a colony formation assay to investigate the long-term effect of CuI on pancreatic cancer lines. The results demonstrated that CuI significantly inhibited the size and number of colonies formed in the BXPC-3 and CFPAC-1 cell lines (Figure 2E-F), suggesting that the antiproliferative effects of CuI are irreversible. CCK-8, EdU and colony formation assays confirmed that CuI inhibits the proliferation of pancreatic cancer.

### Cucurbitacin I suppressed the migration and invasion of pancreatic cancer cells

**Figure 3:**
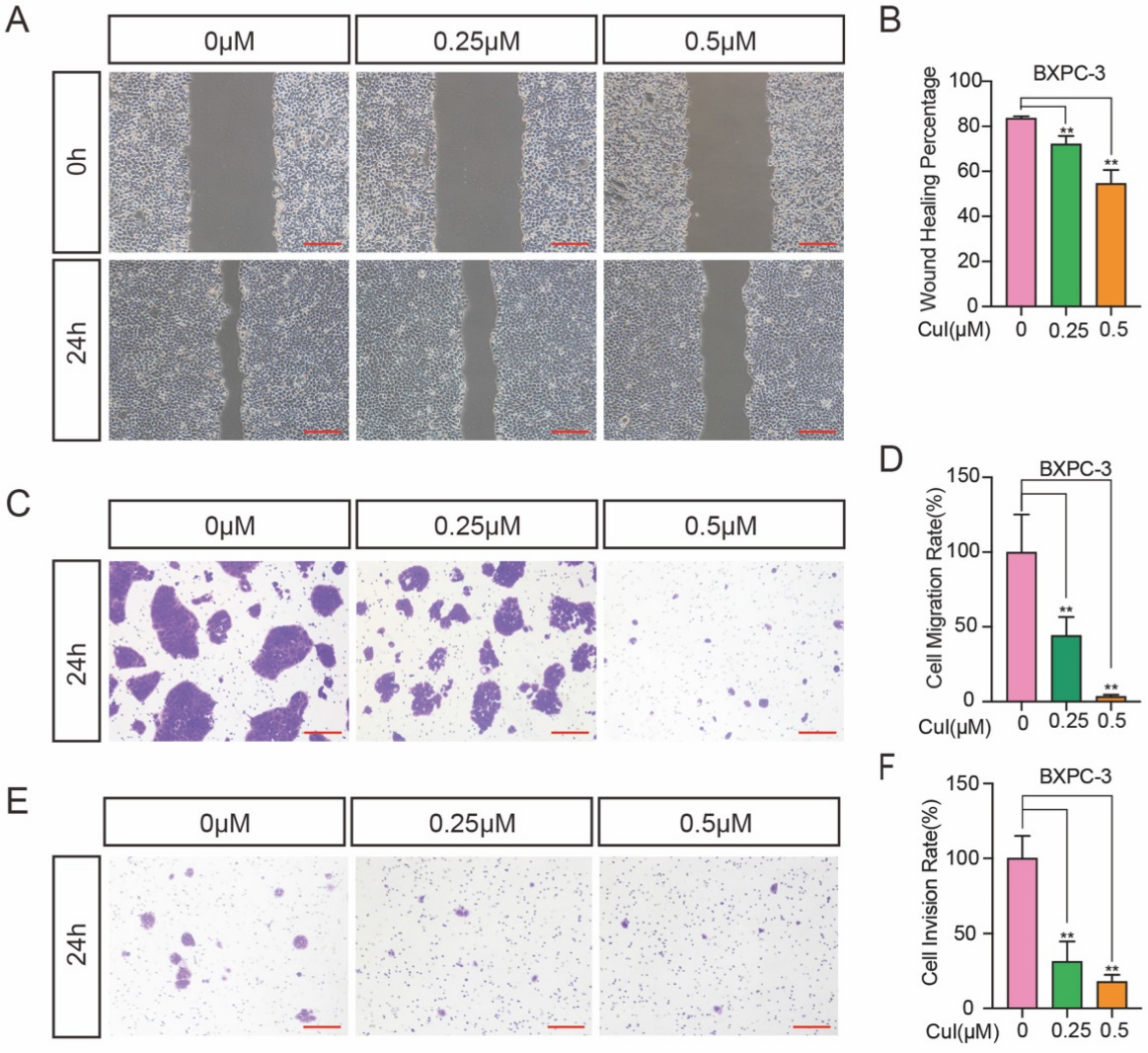
** CuI suppressed the migration and invasion of PDAC. (A)** BXPC-3 cell migration under different concentrations of CuI treatment, scale bar: 200 µm. **(B)** Image J software was used to measure the distance of PDAC migration. **(C)** Transwell assays used to assess cell 3D-migration of BXPC-3, scale bar: 200 µm. **(D)** The area of migrating cells was counted by Image J software. **(E)** Transwell assays with matrix gel used to assess cell migration of BXPC-3, scale bar: 200 µm. **(F)** The area of invasion cells was counted by Image J software.

Wound healing assays and Transwell assays were used to investigate the effects of CuI on 2D and 3D migration and 3D invasion of pancreatic cancer cells. Wound healing involves many processes, including cell proliferation, cell migration and the establishment of cell polarity 20. To limit the influence of cell growth on the wound healing assay, we starved the cells before and during the monolayer cell wound assay. Serum starvation causes the cell cycle arrested, thereby inhibiting cell growth 21. When the wound was still in a static state, wound healing and invasion tests were performed within 24 h, so the diameter of the wound only reflects the migration results. As shown in Figure 3A-B, the migration distance was significantly reduced after CuI treatment, and the migration distance was the shortest in the 0.5 μM group. In addition, we used the Transwell assay without matrix gel for 3D migration detection, and the results were similar to those of the wound healing assay (Figure 3C-D). Since cell invasion is an important feature of pancreatic cancer cells, the decreased percentage of invasive cells passing through the Matrigel coated Transwell membranes indicates that CuI treatment not only reduces the viability of pancreatic cancer cells but also reduces their motility (Figure 3E). Since BXPC-3 and CFPAC-1 cells gather together after passing through the membrane, the number of cells cannot be measured, so we used ImageJ software to calculate the cell area. All experiments in Figure 3 were performed in BXPC-3 and repeated in ASPC-1, CFPAC-1 and SW 1990 (Supplementary Figure 2).

### Cucurbitacin I arrested the pancreatic cancer cell cycle in the G2/M phase and induced cell apoptosis

**Figure 4:**
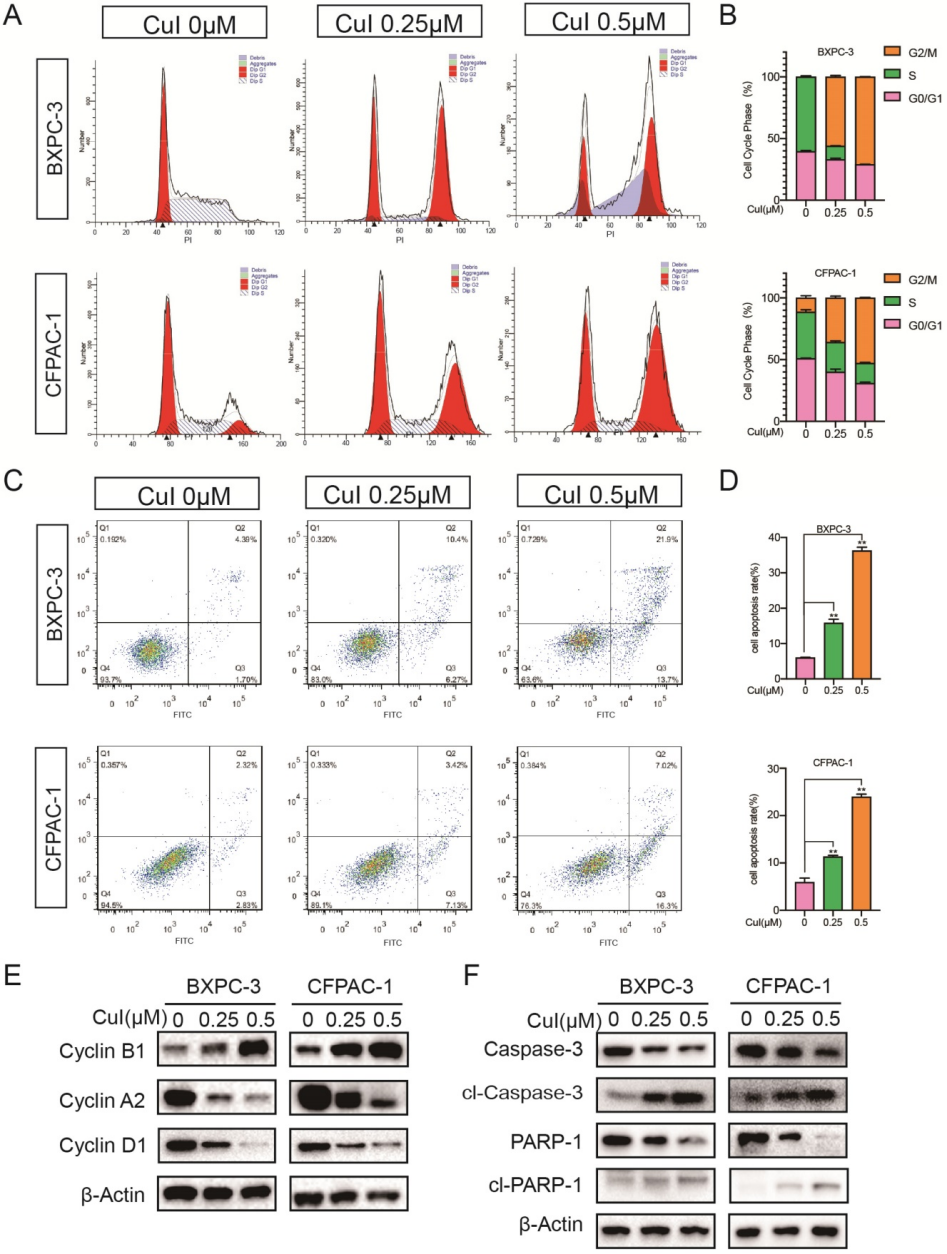
** CuI arrested the PDAC cell cycle in the G2/M phase and induced apoptosis. (A)** FACS analysis of PDAC cell cycle progression after CuI treatment. **(B)** The cell cycle distribution was calculated with Cell Quest Pro software. The cell cycle stagnates in the G2/M phase with the CuI dose. **(C)** Cell apoptosis was assessed with Annexin V-PI staining. **(D)** The group treated with 0.5 µM CuI showed the highest rate of apoptotic cells. **(E)** A decrease of Cyclin D1 and Cyclin A2 protein levels while increase of Cyclin B1 in CuI-treated PDAC cells. **(F)** A decrease of Caspase 3 and PARP1 protein levels while increase of cleaved Caspase 3 and cleaved PARP1 in CuI-treated PC cells.

Previous studies have shown that pancreatic cancer cell proliferation was dose-dependently suppressed by CuI (Figure 1-2). To explore the growth inhibitory mechanisms of CuI, we studied the effect of CuI on the cell cycle control of pancreatic cancer cells by flow cytometry (Figure 4A-B). Compared with that of the control group, CuI treatment increased the cell cycle in the G2/M phase but there was a decrease in S and G0/G phase cells. Furthermore, the induction of apoptosis was examined by Annexin V-PI staining. CuI-treated pancreatic cancer cells showed an enhanced percentage of cells in the early and late apoptotic stages (Figure 4C-D). The western blot results (Figure 4E-F) showed that the expression of cyclin B1 increased significantly under CuI treatment. Since the increase in cyclin B1 is a key sign for cells to enter the M phase, we judged that the cell was stuck in the M phase. In addition, cyclin D1 and cyclin A2 are highly expressed in G1 and S phases, respectively. The decrease of cyclin D1 and cyclin A2 indicated that cell arrest occurred in G2/M phase. The reduction in Caspase3 and PARP1 also confirmed cell apoptosis.

### Cucurbitacin I inhibited the proliferation of pancreatic cancer through the JAK2/STAT3 signalling pathway

**Figure 5:**
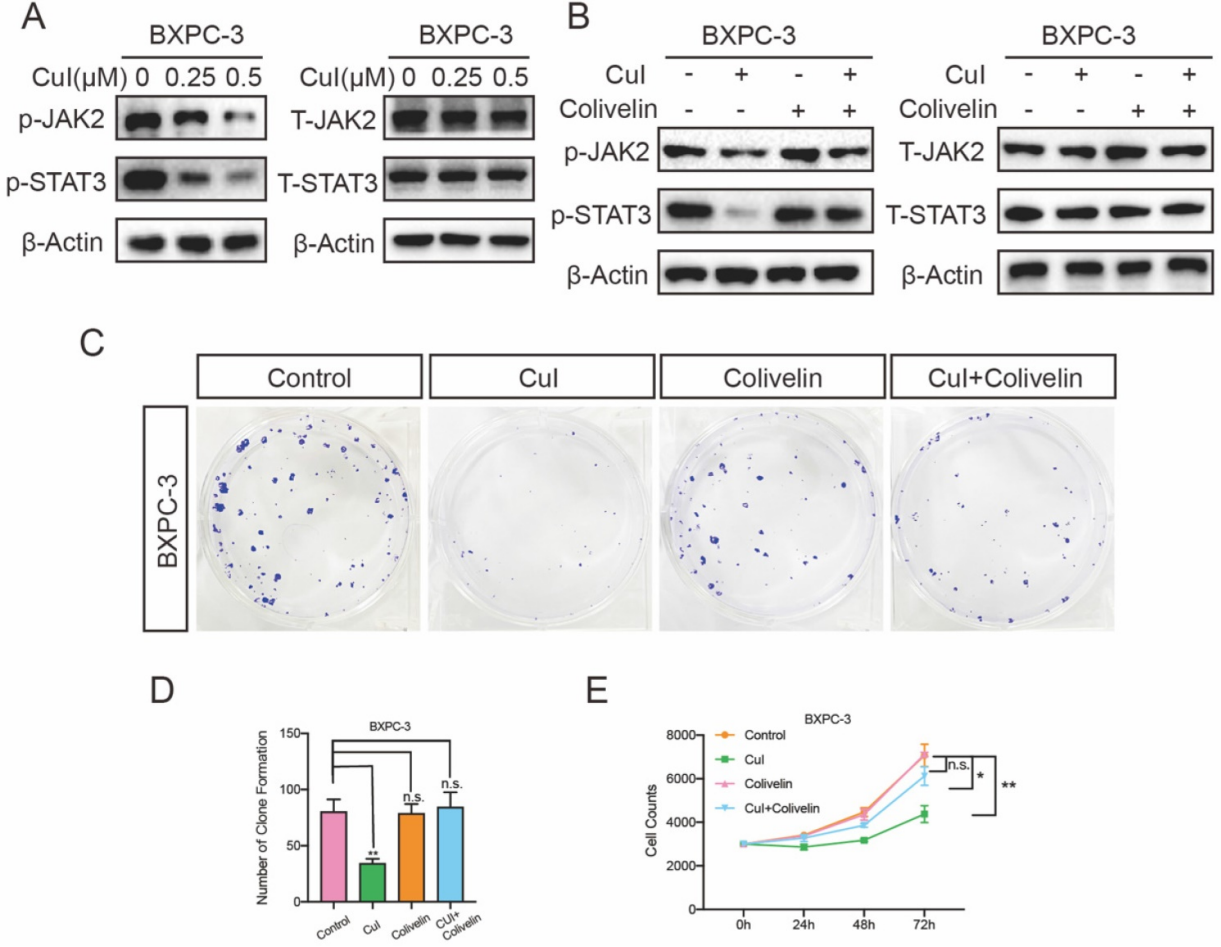
** CuI down-regulated JA2/STAT3 signalling pathway in PDAC cells. (A)** CuI induced a decrease of p-JAK2 and p-STAT3 protein levels and does not influence the protein levels of T-JAK2 and T-STAT3 in CuI-treated PDAC cells. **(B)** Colivelin activated JAK2/STAT3 signalling pathway inhibited by CuI. **(C-D)** Colivelin significantly rescued the number of clones inhibited by CuI. **(E)** Colivelin significantly restored the number of cells inhibited by CuI.

The JAK2/STAT3 pathway plays an important role in cell growth, proliferation and survival and has been associated with many human cancers 22-24. We tested the expression of these proteins after cucurbitacin I treatment. CuI treatment for 5 h significantly inhibited the expression of p-JAK2 and p-STAT3, and interestingly, it did not affect the expression of total protein (Figure 5A). To further determine the JAK2/STAT3 signalling pathway roll in the cucurbitacin-mediated inhibition of pancreatic cancer growth, colivelin, an activator of the JAK2/STAT3 pathway, was used (50 μg/mL). When CuI (0.5 μM) was combined with the signalling pathway activator colivelin, we found that p-JAK3 and p-STAT3 expression was restored, confirming the successful role of colivelin (Figure 5B). On this basis, we conducted clone formation experiments and found that the size and number of colonies were significantly improved. In addition, we measured the effect on cell proliferation, which was also significantly improved after restoration of the JAK2/STAT3 signalling pathway. These results indicate that CuI inhibits cell proliferation by supressing the JAK2/STAT3 signalling pathway. All experiments in Figure 5 were performed in BXPC-3 cells and repeated in CFPAC-1 cells (Supplementary Figure 3).

### Cucurbitacin I treatment inhibited pancreatic tumour growth *in vivo*

To determine whether CuI treatment can suppress pancreatic tumour growth *in vivo*, we conducted an orthotopic xenograft study. Different doses of CuI were injected every three days, and the body weight and tumour volume of the mice were recorded. The experiment ended after 30 days, and the tumour volume of the mice treated with CuI was significantly smaller than that of the control group. At the same time, the tumour volume of the mice treated with higher doses (2 mg/kg) was smaller than that of the mice treated with lower doses (1 mg/kg). In addition, we found that the weight of the mice treated with CuI was generally heavier than that of the control group but it was not statistically significant, and the weight of the mice still increased throughout the experiment. Therefore, we judged that the treatment had no obvious negative effects. After removing the tumour, we performed western blotting and immunohistochemical experiments on the tumours of the mice to test the expression of STAT3 and PCNA, which represents tumour proliferation. The western blot experiments found that the expression of p-STAT3 in the tumours of mice was significantly reduced after CuI treatment. Immunohistochemical experiments found that the control group had the highest expression of PCNA. The results of the above experiments are consistent with our *in vitro* research.

## Discussion

In the past few years, many great advances have been made in understanding the molecular changes in pancreatic cancer and the prognostic significance of these changes. Pancreatic cancer is well known to be an oncogene-driven tumour with a variety of genetic and epigenetic changes. Almost all patients with pancreatic cancer carry at least one of the known driving genes that are frequently mutated, oncogene K-RAS and tumour suppressor genes CDKN2A, TP53 and Smad4/DPC4 25, 26. The loss of TP53 function induces the activation of JAK2/STAT3 signalling, which can promote tumour growth and tumour resistance to gemcitabine 27. In this study, we explored the anti-pancreatic cancer effect of CuI, an extract of the Cucurbitaceae family. This is the first evidence that CuI inhibits the proliferation of pancreatic cancer cells by suppressing JAK2 and STAT3 signal transduction.

Cucurbitacin, as a traditional anti-inflammatory and analgesic gene, has been used for centuries in China and other Eastern countries. In recent years, there have been many reports on the possibility of cucurbitin as a treatment for malignant tumours. Cucurbitacin B has been shown to induce cell cycle arrest, apoptosis, and autophagy 28, 29. Cucurbitacin IIA may cause irreversible actin aggregation 30. Cucurbitacin E inhibits cellular proliferation and enhances the chemo-response in gastric cancer 31. In addition, CuI has been shown to play a role in a variety of tumours 32-34. Our study also showed that CuI exerts an anti-tumour effect in pancreatic cancer cells.

Metastasis is the main cause of incurable malignancies and one of the characteristics during cellular migration and invasion 35. Our wound healing and Transwell assays showed that CuI significantly inhibited the invasion and migration of pancreatic cancer cells, which indicated that CuI may be an effective compound for inhibiting the metastasis of pancreatic cancer cells. However, more detailed studies are needed to investigate the effects of cucurbitin D treatment on the metastatic growth of pancreatic cancer cells in an appropriate mouse model of pancreatic cancer.

Treatment of human pancreatic cancer xenograft nude mice with CuI significantly delayed tumour growth. Regarding the toxicity of CuI in the body, there were no obvious signs of poisoning, including no significant changes in body weight, with three days of treatment. This is consistent with previous research, compared with normal cells, CuI is selectively cytotoxic to cancer cells 36. We speculate that this may be related to abnormal JAK2/STAT signalling pathway activation in pancreatic cancer. Consistent with our *in vitro* observations, the expression of activated STAT3 in the tumours of these mice was reduced compared to that in the control group. In addition, immunohistochemistry showed that the expression of the proliferation-related protein PCNA in the tumour gradually decreased in the control group, and that of the low-dose and high-dose groups gradually decreased.

In conclusion, our research shows that CuI has significant anti-pancreatic cancer activity both *in vivo* and *in vitro*. The compound inhibits the JAKT/STAT pathway and induces cell cycle arrest and apoptosis in pancreatic cancer cells. Our research provides a theoretical basis for the development of cucurbitacin I as a drug for the treatment of pancreatic cancer.

## Supplementary Material

Supplementary figures.

## Figures and Tables

**Figure 6:**
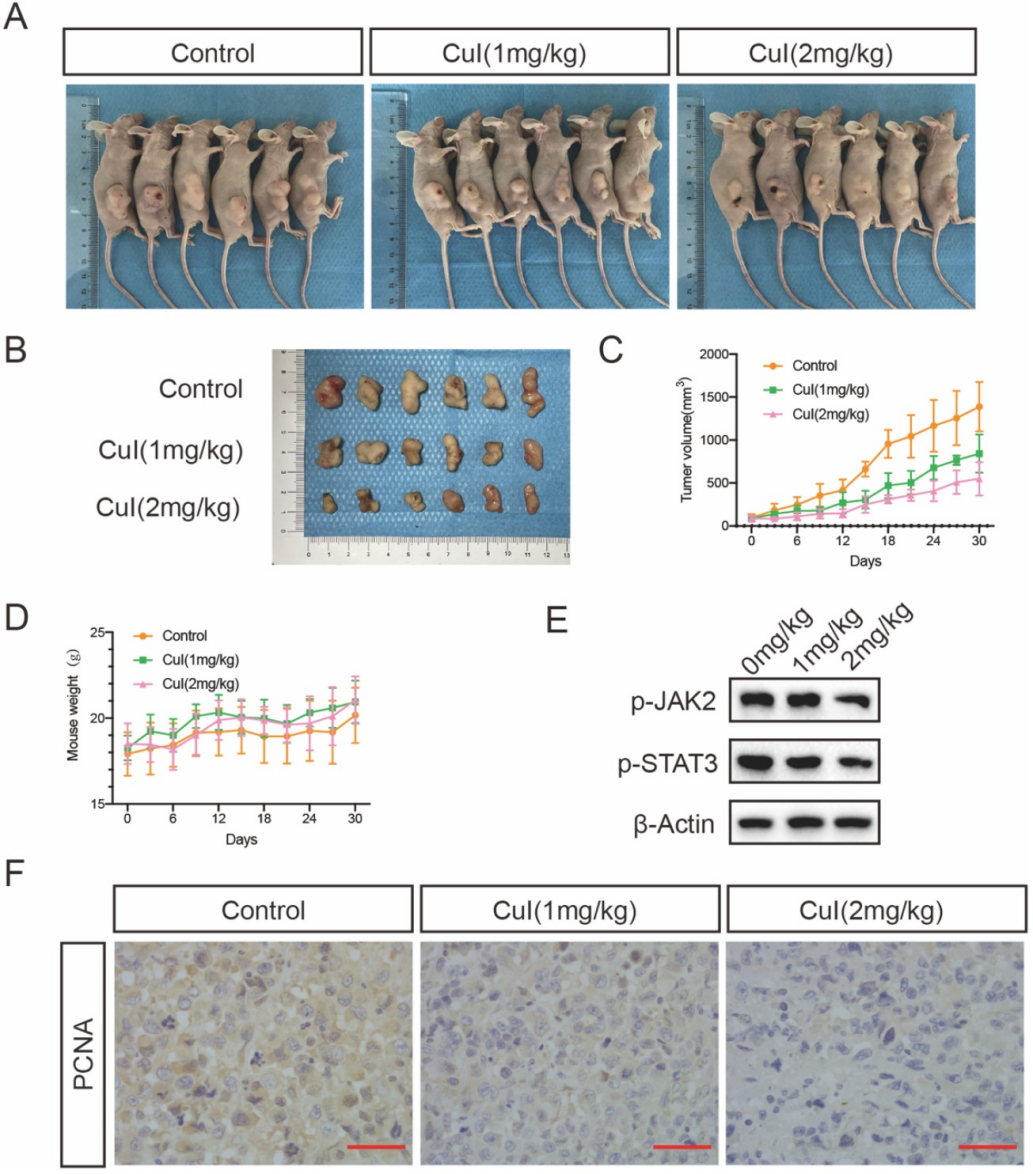
** CuI inhibited the growth of PDAC tumor xenografts *in vivo*. (A)** Images of control group, low-dose (CuI 1mg/kg) and high-dose (CuI 1mg/kg) tumor-bearing mice. **(B)** Tumor masses from the control group, from the low-dose group and the high-dose group. **(C)** Measured the length and width every 3 days to calculate the volume of the tumor from day 0 to day 30 after injected two weeks. **(D)** Measured the weight of mice every 3 days. **(E)** CuI induced a decrease of p-JAK2 and p-STAT3 protein levels in mice tumor. **(F)** The expressions of PCNA in xenograft tumors were analyzed by immunohistochemistry.
