# Supplementary material for: Cucurbitacin I inhibits the proliferation of pancreatic cancer through the JAK2/STAT3 signalling pathway in vivo and in vitro
Source: J Cancer. 2022 Mar 28;13(7):2050–60. doi: 10.7150/jca.65875 (PMC9066209; doi:10.7150/jca.65875)
Supplement: Supplementary file 1 — Supplementary figures. [file jcav13p2050s1.pdf]

Supplementary Material

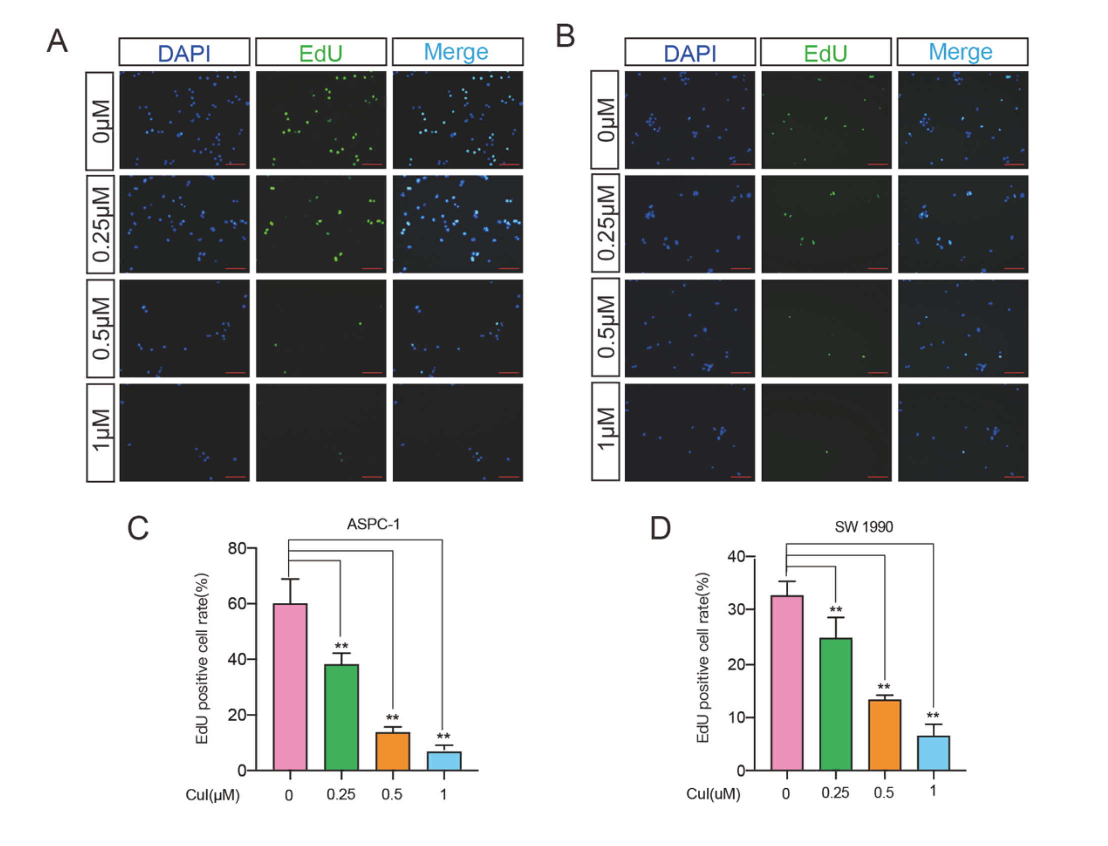

Supplementary Figure 1

CuI inhibited the proliferation of PDAC cells in ASPC-1 and SW 1990. (A-B) Detection by fluorescence microscopy of EdU (green) incorporated into the DNA of cultured ASPC-1 and SW 1990 cells, scale bar: 200 μm. The nuclei were counter-stained with DAPI (blue). (C-D) The EdU positive rate of ASPC-1 and SW 1990 cells.

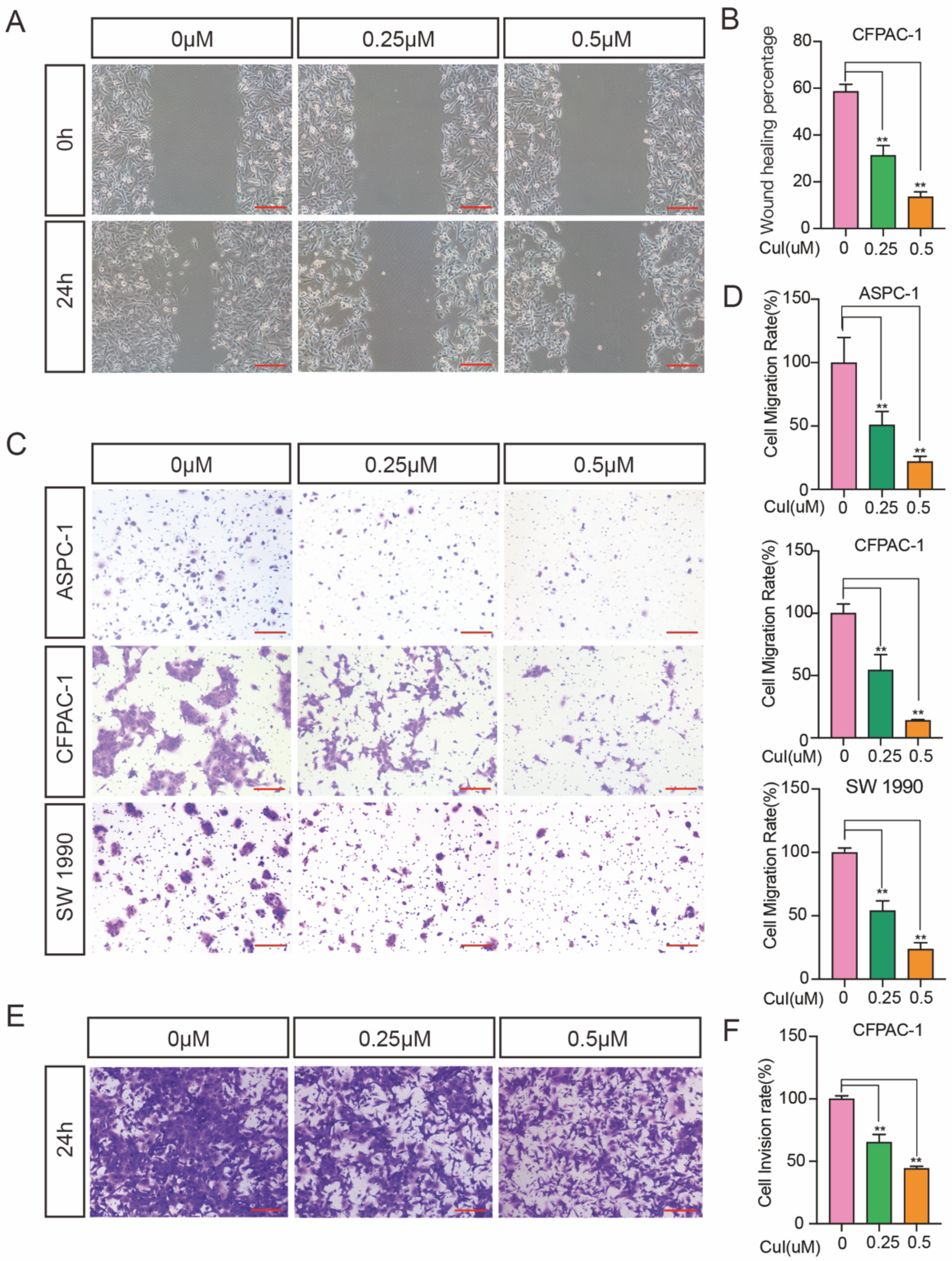

Supplementary Figure 2

CuI suppressed the migration and invasion of CFPAC-1 cells (A) CFPAC-1 cell migration under different concentrations of CuI treatment, scale bar: 200 μm. (B) Image J software was used to measure the distance of PDAC migration. (C) Transwell assays used to assess cell 3D-migration of ASPC-1, CFPAC-1 and SW1990, scale bar: 200 μm. (D) The area of migrating cells is counted by Image J software. (E) Transwell assays with matrix gel used to assess cell migration of CFPAC-1, scale bar: 200 μm. (F) The area of invasion cells is counted by Image J software.

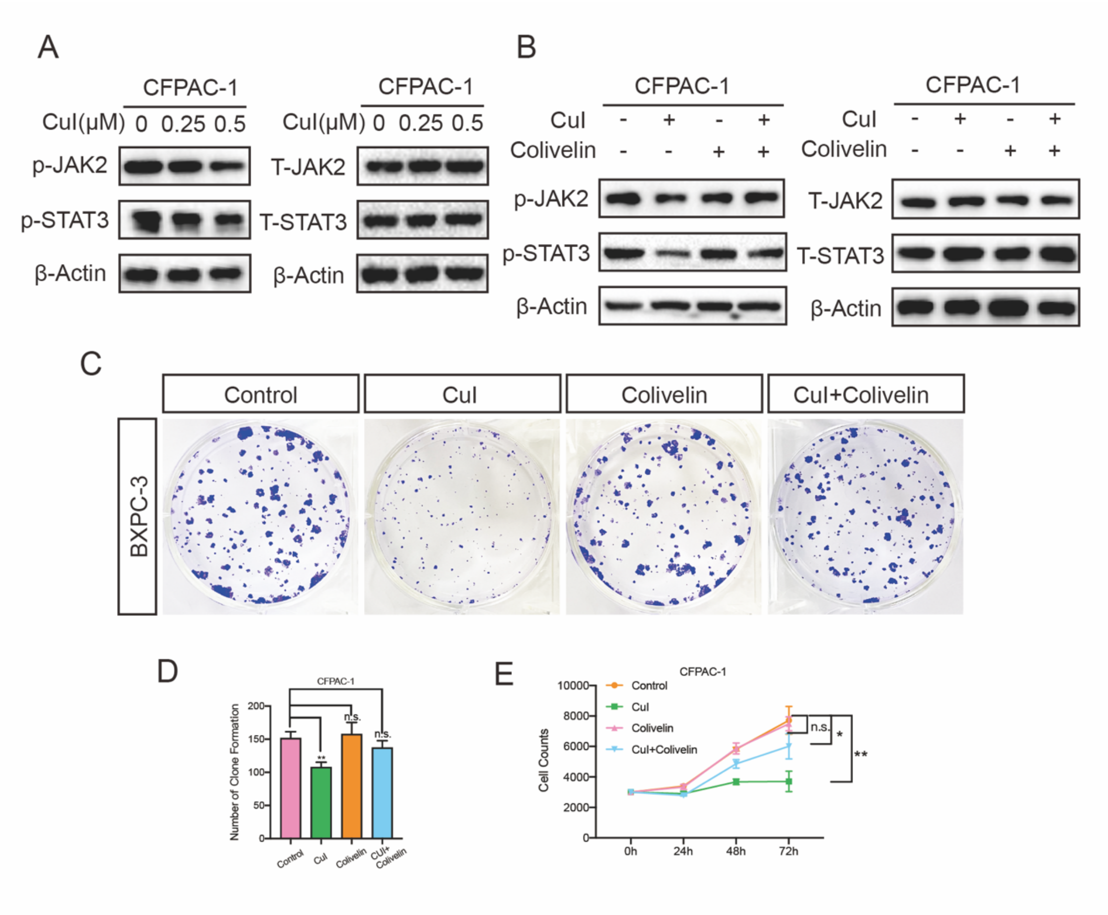

Supplementary Figure 3

CuI down-regulates JA2/STAT3 signaling pathway in CFPAC-1 cells. (A) CuI induced a decrease of p-JAK2 and p-STAT3 protein levels and does not influence the protein levels of T-JAK2 and T-STAT3 in CuI-treated PDAC cells. (B) Colivelin activates JAK2/STAT3 signaling pathway inhibited by CuI. (C-D) Colivelin significantly rescue the number of clones inhibited by CuI. (E) Colivelin significantly restores the number of cells inhibited by CuI.
